# Oral microbiome and preterm birth

**DOI:** 10.3389/fmed.2023.1177990

**Published:** 2023-08-07

**Authors:** Marijana Vidmar Šimic, Aleš Maver, Ana Nyasha Zimani, Keli Hočevar, Borut Peterlin, Anja Kovanda, Tanja Premru-Sršen

**Affiliations:** ^1^Division of Obstetrics and Gynecology, Department of Perinatology, University Medical Centre Ljubljana, Ljubljana, Slovenia; ^2^Clinical Institute of Genomic Medicine, University Medical Centre Ljubljana, Ljubljana, Slovenia; ^3^Faculty of Medicine, University of Ljubljana, Ljubljana, Slovenia

**Keywords:** preterm delivery, microbiome, 16S rRNA gene, 16S rDNA, oral microbiome, pregnancy

## Abstract

**Background:**

The etiology of preterm birth (PTB) is heterogeneous and not yet well known. Maternal periodontal disease has been investigated for decades and is a known risk factor for adverse pregnancy outcomes. However, no particular bacterial species or higher taxonomic order has been found as causative of PTB, leading to studies of the whole oral microbiome. In order to determine if and how the composition of the oral microbiome is associated with PTB, we performed a large case–control study including women with term (TB) and PTB.

**Methods:**

We compared oral microbiomes in PTB to TB, to examine differences in the microbial richness, diversity, and differential abundance of specific taxa. We obtained oral swab samples from 152 Caucasian pregnant women who were classified as either PTB (≤36 6/7 weeks, *n* = 61) or TB (≥38 0/7 weeks, *n* = 91) in exclusion of any other major medical or obstetric conditions. The oral microbiomes of these women were characterized by 16S ribosomal RNA (rRNA) gene sequencing of the V3–V4 region on the MiSeq platform.

**Results:**

The dominant microorganisms at the phylum level in all pregnant women regardless of birth week outcomes as belonging to *Firmicutes, Proteobacteria, Bacteroidetes, Fusobacteria*, and *Actinobacteria*. The phyla *Firmicutes* and *Bacteroidetes* were relatively more abundant in women with a PTB than in women with a TB, while *Proteobacteria* was less prevalent in women with a PTB. At the genus level, *Veillonella*, *Prevotella*, and *Capnocytophaga* were enriched in the PTB, and while many of the members of these genera could not be resolved to the species level, *Veillonella massillensis* was shown to be increased in the PTB group.

**Conclusion:**

We identified the genera *Veillonella*, *Prevotella*, and *Capnocytophaga* in the maternal oral microbiome as being associated with PTB independently of clinically apparent infection, uterine anomalies, and other pregnancy complications, including placenta previa, and placental abruption. The clarification of the role of those taxa in the etiology of PTB merits further research.

## 1. Introduction

A normal pregnancy lasts approximately 40 weeks, while deliveries before 37 weeks are considered preterm. Every year, an estimated 15 million babies are born preterm (1, 2). Infants born preterm are at high risk of neonatal mortality and morbidities, which potentially affect childhood development and may cause long-term health problems throughout their lives.

The etiology of preterm birth (PTB) is heterogeneous and not yet well known. In about one-third of cases, PTB is iatrogenic. In other instances, PTB occurs after the spontaneous onset of preterm labor due to various causes, including spontaneous contractions, preterm premature rupture of membranes (PPROM), intrauterine infection, cervical insufficiency, and others (3). PTB is also influenced by associated factors such as smoking, substance abuse, mental health problems, genetics, and physical overexertion (4). The origins of infections and inflammation, which may be the cause of PTB, have so far been associated with intrauterine (chorioamnionitis) and extra-uterine maternal infections, such as kidney infection, vaginal infection, and periodontal disease (5–7).

In terms of the association between PTB and oral health, maternal periodontal disease has been investigated for decades as a risk factor for adverse pregnancy outcomes. In a systematic review and meta-analysis including over 10,000 pregnant women, it was reported that women with periodontal disease during pregnancy are twice as likely to have PTB (8).

Following this association, research began on the role of oral bacteria in association with PTB. As summarized in a recent meta-analysis, different studies (cultivation and non-cultivation methods) have linked oral microbiota composition to PTB (9). However, due to the differences in study design, approach to bacterial detection (direct, targeted, culturing vs. sequencing), and small numbers of included samples, they have not been able to consistently implicate a particular bacterial species or higher taxonomic order as causative of PTB (9–12).

Therefore, in order to evaluate the possible contribution of the composition of the oral microbiome in PTB, we performed an independent case–control study on the Slovenian population of pregnant women with term and PTB, by using 16S rDNA sequencing.

## 2. Materials and methods

### 2.1. Ethics statement

The study was approved by the Slovenian National Medical Ethics Committee (approval number 90/02/15) and was conducted according to the principles in the Declaration of Helsinki. All women included in the research provided written informed consent before recruitment.

### 2.2. Sample collection and clinical assessment

The study was conducted at the Department of Perinatology of the University Medical Centre Ljubljana, Slovenia, between October 2015 and February 2020. One hundred and seventy-three (173) singleton pregnant women were recruited initially to the study. The week of pregnancy was defined according to each woman’s last menstrual period and confirmed through ultrasound examination in the first trimester by measuring the fetal crown-rump length (13). Oral swabs were collected upon admission to the hospital due to signs of preterm/term labor or just before planned cesarean sections. The samples from the maternal oral cavity consisted of a pooled saliva, tongue, hard palate, and buccal mucosa swab. The inclusion criteria were a singleton pregnancy and gestational age of 22–42 weeks at delivery. The included women were over 18 years of age, and were able to provide informed consent. We excluded women who had had multiple pregnancies, stillbirths, chronic hypertension, gestational hypertension (incl. preeclampsia), pre-pregnancy diabetes mellitus, uterine anomalies, and other pregnancy complications, including placenta previa, placental abruption, and pregnancies with congenital anomalies or fetal growth restriction (FGR). We excluded women who had used antibiotics for any type of infection during the week before inclusion, those who were receiving immunosuppressive therapy, or had human immunodeficiency virus (HIV) or hepatitis C-positive status.

The study and control groups were defined according to gestational age and the beginning of delivery. The study group was defined as spontaneous onset of premature delivery with or without PPROM before or at 36 6/7 weeks of gestation (PTB group), and the control group was defined as spontaneous onset of delivery with or without PROM or planned cesarean section at or after 38 0/7 weeks of gestation (TB group).

Participants were also asked to complete a brief in-person questionnaire to collect additional demographic and medical information (age, working status, cigarette smoking, pre-pregnancy BMI (body mass index), antibiotics use during pregnancy, gestational diabetes mellitus). The National Perinatal Information System of Slovenia (NPIS) was reviewed to collect additional data on the pregnancy, delivery, and postpartum period.

### 2.3. Bacterial DNA isolation, 16S rRNA gene amplification and sequencing, and bioinformatics analysis

The oral swabs for genomic bacterial DNA isolation were transferred to the laboratory in the Amies transport medium and stored at −80°C until isolation.

Isolation was performed as previously described (14). Briefly, mechanical and enzymatic lysis using lysozyme was performed as described by Ravel et al. (15). Bacterial DNA was obtained from the lysate swab medium using QIAamp DNA Mini Kit (QIAGEN, Hilden, Germany) according to the manufacturer’s instructions. DNA concentrations were measured using a Qubit dsDNA high-sensitivity assay kit (Thermo Fisher Scientific).

Bacterial 16S rDNA was amplified using PCR with an Illumina adapter containing primers 341F and 805R for the informative V3–V4 region, and the PCR products were visualized on an agarose gel before proceeding to clean-up and indexing.

The amplicons of each sample were labeled with Nextera XT Indexes, and sequencing libraries were prepared according to the standardized 16S Metagenomic Sequencing Library Preparation Protocol (Illumina®). Libraries of pooled samples were sequenced on the Illumina MiSeq sequencer according to the manufacturer’s specifications in the 2 × 300 bp pair-end runs (MiSeq Reagent kit v3).

### 2.4. Bioinformatics analysis

Bioinformatics analyses were performed in R environment version 4.2. using the Bioconductor workflow adapted from Callahan et al. (16).

Briefly, sequence data consisting of demultiplexed FASTQ files with at least 30 Phred scores were trimmed and filtered using DADA2 (1, 17). Trimming was performed on joint reads so that the first 17 forward reads and the first 21 reverse reads were trimmed to remove primers, and all reads were truncated to 250 in length. No ambiguous base calls were allowed, and filtering parameters were set to a maximum of 2 expected errors per read pair. Quality scores and error rates were assessed separately for all runs to minimize batch effects resulting from run variability. All runs contained a balanced representation of pre-term and term samples.

Amplicon sequence variants (ASV) were inferred, and chimeric sequences were removed using DADA2 (17, p. 2), (18). Taxonomical classification of amplicon sequence variants was determined using the RDP version 18 (19) and Silva v138.1 (20). Sequence, taxonomic and clinical data were combined into a single object using the phyloseq package for R (version 1.40.0) (21).

### 2.5. Statistical analysis

A comparison of demographic and clinical characteristics of PTB and TB was performed using an unpaired t-test for parametric continuous variables and the Mann–Whitney *U*-test for nonparametric continuous variables. An χ^2^ test was performed to test for statistically significant differences between the groups.

Statistical analyses of the microbiome were performed in R version 4.2. using the vegan package version 2.6-2 (22) and DESeq2 version 1.36 (23, p. 2). The prevalence threshold was set at 5% of the total samples. Rarefaction without replacement was performed to even depth (24). The microbial richness and alpha diversity of the sample groups were visualized using Chao1 and the Simpson diversity index, as implemented in the phyloseq package (version 1.40.0) (21). Beta diversity was visualized by generating Principal Coordinate Analysis (PCoA) using unweighted UniFrac as distance in the phyloseq and ggplot2 package for R (21, 25).

The differential relative abundance of ASV and corresponding taxonomic groups was calculated using DESeq2 version 1.36 (23, p. 2). Samples were normalized using the Wald test, and the default Benjamini-Hochberg correction implemented in the DESeq function was used for multiple-inference correction.

## 3. Results

### 3.1. Characteristics of participants

Initially, 173 pregnant women were recruited for the study. Eleven (11) cases were excluded after the medical records were reviewed because they were included in the PTB group (threatened preterm delivery) but subsequently delivered at term, and 10 cases were excluded because they delivered at another hospital.

Of the 152 women analyzed in the study, 61 were in the PTB group, with spontaneous onset of premature labor with or without PPROM (≤36 6/7 weeks), and 91 were in the TB group, with spontaneous onset of labor with or without PROM or planned cesarean section (≥38 0/7 weeks; term—control group).

Out of the 152 included participants, nine delivered with an elective cesarean section (5.9%), while all others had a vaginal delivery (94.1%). In Table 1, the demographic and clinical characteristics of participants are presented. The PTB and TB groups did not differ significantly in terms of age, pre-pregnancy BMI, parity, gestational diabetes mellitus, working status, smoking, marital status, fetal sex, gynecological surgeries, presence of infections such as urogenital infection, asymptomatic bacteriuria and antibiotic use during pregnancy. All included participants were Caucasian.

**Table 1:** Demographic and clinical characteristics.

Demographic and clinical characteristics	Preterm birth ≤ 36 6/7 weeks	Term birth ≥ 38 0/7 weeks	*p*-value, χ^2^ test, ^*^Mann–Whitney test
Total (*N* = 152)	*N* = 61	*N* = 91	
**Maternal age at delivery, years; Mean ± SD**	32.24 ± 4.51	31.40 ± 4.03	*P* = 0.151^*^
**Pre-pregnancy BMI (kg/m^2^) mean ± SD**	23.95 ± 4.96	23.80 ± 4.33	*P* = 0.717^*^
Underweight (<18.5)	4 (6.6)	10 (6.6)	*P* = 0.075, χ^2^ = 6.901
Normal Weight (18.5-24.9)	41 (67.2)	52 (57.1)	
Overweight (25–29.9)	6 (9.8)	24 (26.4)	
Obese (>30)	10 (16.4)	9 (9.9)	
**Birthweight (g; mean ± SD)**	1,852 ± 761	3,477 ± 455	*P* < 0.0001*
**Parity**			*P* = 0.08, χ^2^ = 5.061
0	31 (50.8)/61	30 (33.0)/91	
1	24 (39.3)/61	44 (48.4)/91	
2–4	6 (9.8)/61	16 (17.6)/91	
**Gestational diabetes mellitus**	12 (19.7)	9 (9.9)	*P* = 0.098, χ^2^ = 2.935
**Working status—employed**	48 (80.0)	73 (81.1)	*P* = 1.000, χ^2^ = 0.028
**Smoking**			*P* = 0.497, χ^2^ = 3.377
Yes	7 (10.5)	9 (9.9)	
No	52 (85.2)	77 (84.6)	
Quit before pregnancy	2 (3.3)	5 (5.5)	
**Marital status—in partnership**			*P* = 0.326, χ^2^ = 2.243
**Fetal sex**	60 (98.4)	91 (100)	*P* = 0.409, χ^2^ = 0.800
Female	25 (41.0)	44 (48.4)	
Male	36 (59.0)	47 (51.6)	
**Gynecological surgeries**	18 (29.5)	16 (17.6)	*P* = 0.084, χ^2^ = 2.991
**Previous PTB**	7 (11.5)	3 (3.3)	***P* = 0.046, χ**^**2**^ **= 3.975**
**Bleeding before 14th week of gestation**	24 (40.7)	17 (18.9)	***P* = 0.005, χ**^**2**^ **= 8.483**
**Urogenital infection**	5(8.5)	8 (8.9)	*P* = 1.000, χ^2^ = 0.008
**Asymptomatic bacteriuria**	1 (1.7)	3 (3.3)	*P* = 1.000, χ^2^ = 0.366
**Antibiotic use during pregnancy**	4 (6.6)	1 (1.1)	*P* = 0.152, χ^2^ = 3.764
**Tocolytic therapy (Atosiban)**	4 (6.6)	0 (0.0)	***P* = 0.013, χ**^**2**^ **= 6.128**

Demographic and clinical characteristics in preterm and term deliveries. Parametric continuous variables were tested using an unpaired *t*-test and are given as means ± standard deviations; nonparametric continuous variables were tested using the Mann–Whitney *U*-test. BMI, body mass index; PTB, preterm birth; TB, term birth. Statistically significant differences between the PTB and TB groups are given in bold. *Mann-Whitney test.

Women with prior PTB and with vaginal bleeding before the 14th week of gestation in the current pregnancy were more likely to experience PTB (*p* = 0.046 and *p* = 0.005, respectively), as is already well known from previous reports (26). Of the PTB group, 25 (41%) women experienced PPROM. As expected, in the PTB group newborns’ birthweights were lower and tocolytic therapy was used more often than in the TB group.

### 3.2. 16S sequencing results

V3-V4 region 16S rRNA sequencing (MiSeq, Illumina) was performed on a total of 152 samples (61 PTB and 91 TB group).

MiSeq sequencing generated a total of 23,290,100 raw reads that, after quality control and chimera removal, resulted in a total of 11,568,634 processed paired-end V3-V4 reads, with an average read count of 76,109 reads per sample (min = 30,154; max = 152,716). Our analyses identified the presence of >10,000 ASVs. After the removal of singletons, doubletons, rare ASV (occurring in less than 5% of samples), and unassigned taxa, sequences could be assigned to 2,465 OTU (operational taxonomic units) in 152 samples. Of the identified 2,465 OTUs, 375 could be resolved at least to the genus level.

### 3.3. Diversity and composition of the oral microbiome

**Figure 1:**
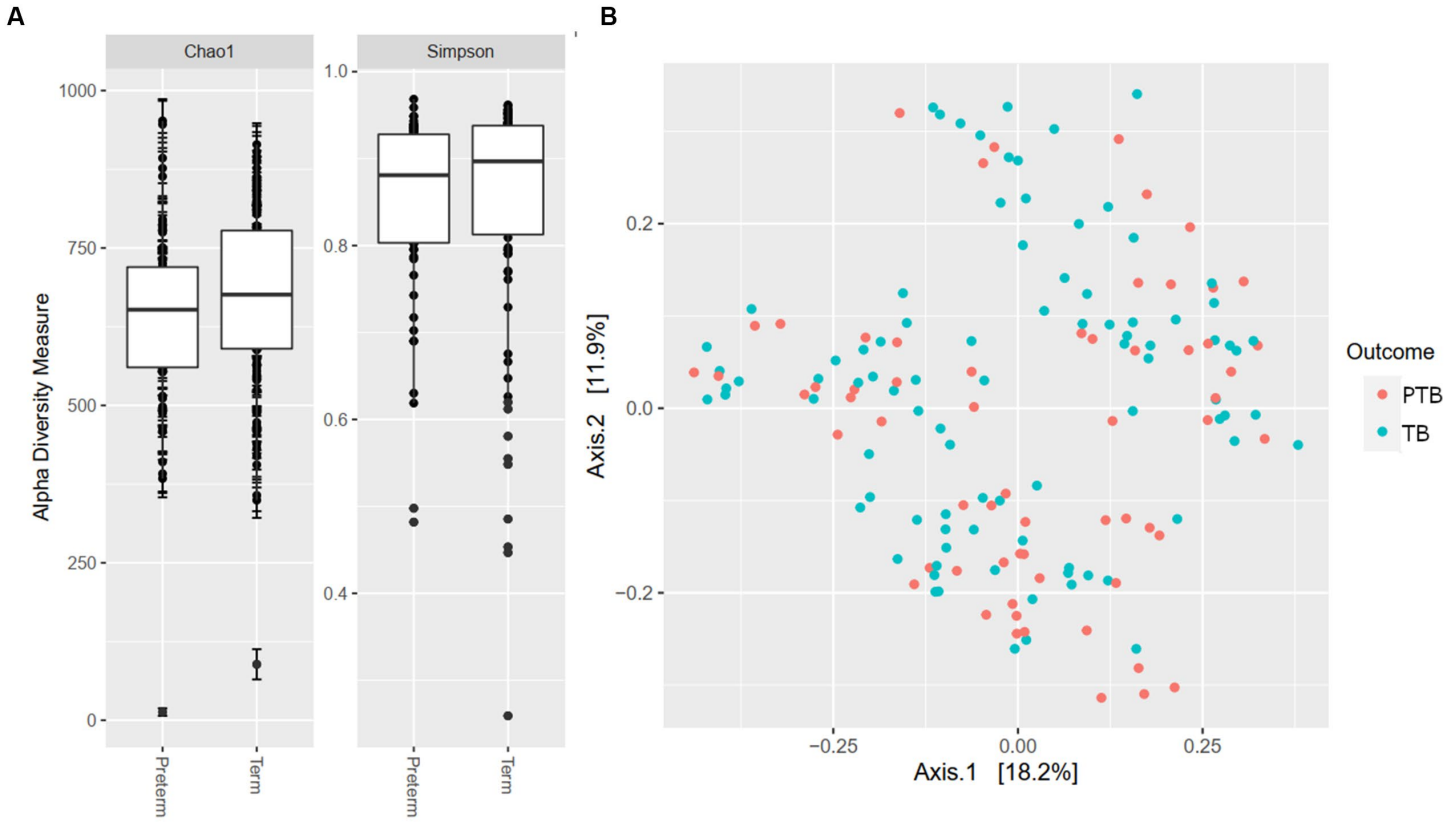
Alpha and beta diversity by gestational delivery groups. **(A)** Alpha diversity of preterm and term birth groups. **(B)** Principal coordinate analysis (PCoA) plot generated by the Weighted Unifrac distance between preterm (PTB) and term birth (TB) groups.

Our analyses identified the presence of >10,000 ASV that could, after removal of rare sequences be assigned to 375 known taxa to the level of genera, showing high oral microbiome diversity in pregnant women. When examining microbial richness using alpha and beta diversity measures, there were no significant differences between the PTB and TB groups (Figures 1A,B).

**Figure 2:**
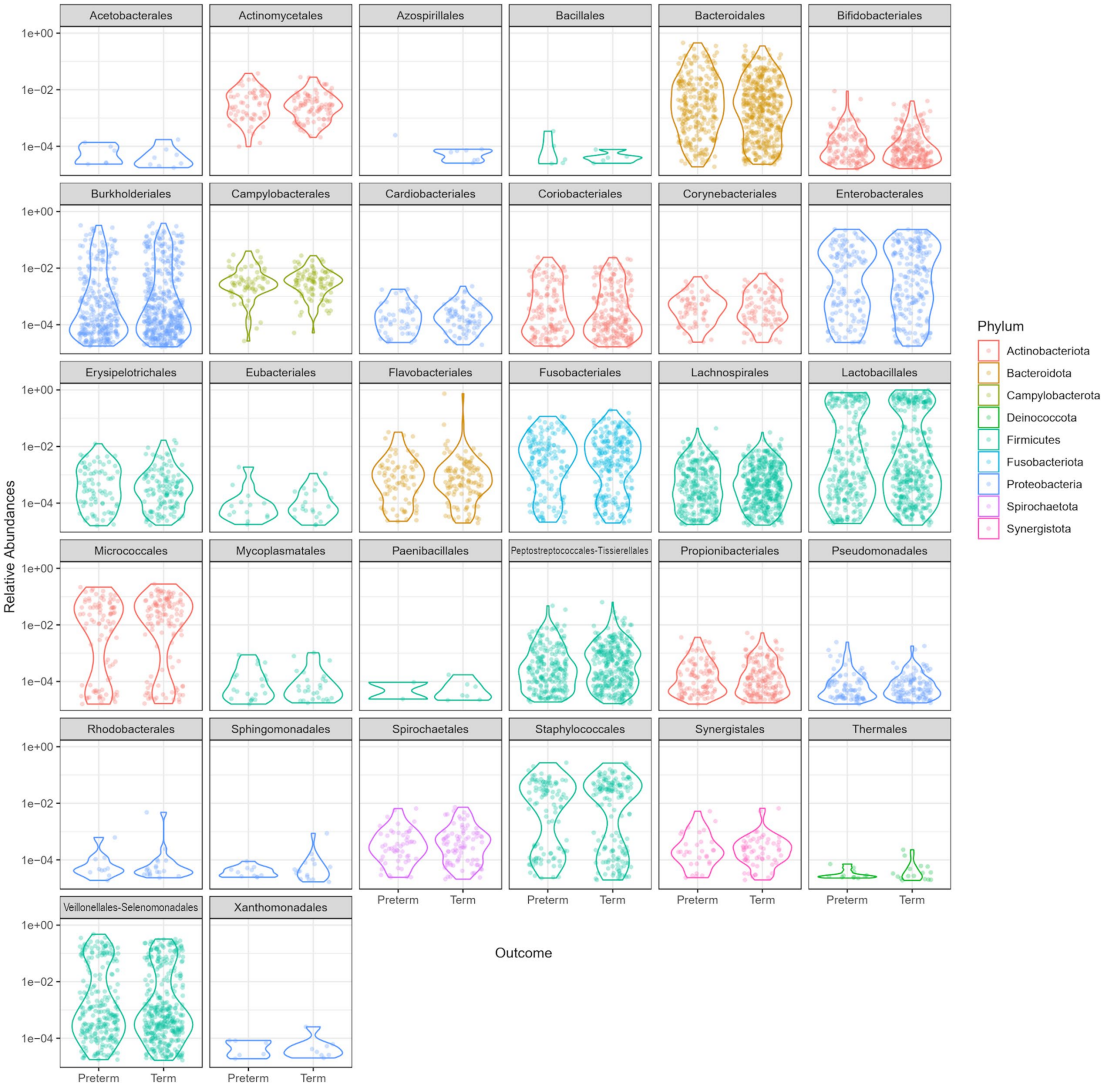
Relative abundance comparison of phyla in the oral microbiome of the preterm (PTB) and term birth (TB) groups.

Overall, a similar relative abundance of bacterial phyla was observed in the oral microbiome of the PTB and TB groups (Figure 2).

The dominant bacterial phyla in the oral microbiome of pregnant women included in the study were *Firmicutes, Proteobacteria* (*Pseudomonadota*)*, Bacteroidetes, Fusobacteria*, and *Actinobacteria* (Figure 2).

**Figure 3:**
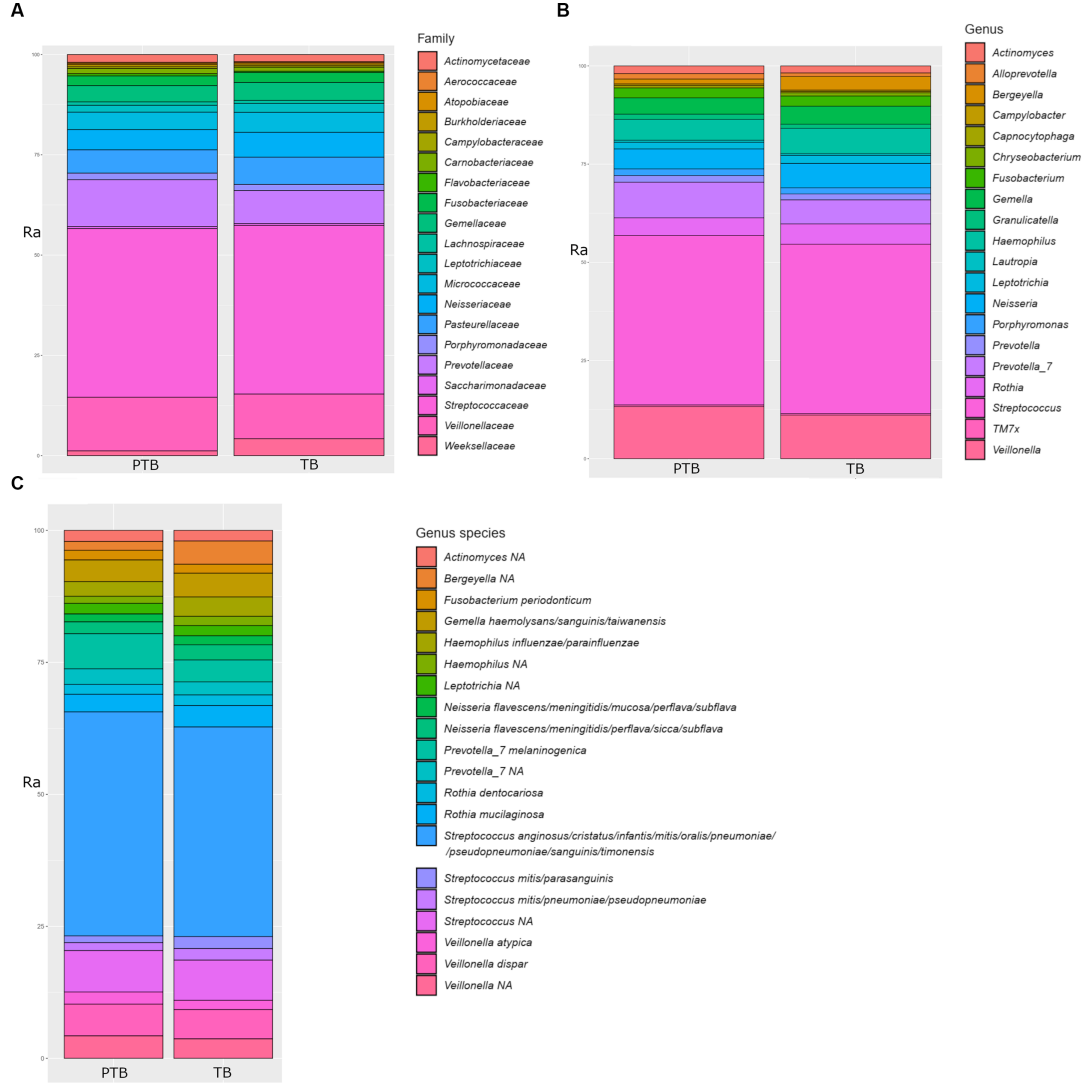
Comparison of top 20 oral microbial families, genera, and species in the preterm and term delivery groups. **(A)** Comparison of top 20 bacterial families in the oral microbiome of the preterm (PTB) and term birth (TB) groups. **(B)** Comparison of top 20 bacterial genera in the oral microbiome of the preterm (PTB) and term birth (TB) groups. **(C)** Comparison of top 20 bacterial species in the oral microbiome of the preterm (PTB) and term birth (TB) groups. Ra, relative abundance in %.

The family *Streptococcaceae* represented 39.50% of total oral bacteria, followed by *Veillonellaceae* 11.45%, *Prevotellaceae* 9.55%, *Pasteurellaceae* 6.25%, *Neisseriaceae* 5.68%, *Microccaceae* 4.51%, *Gemellaceae* 4.06%, *Weeksellaceae* 2.82%, *Fusobacteriaceae* 2.31%, *Leptotrichiaceae* 2.31%, *Leptotrichiaceae* 1.91%, *Actinomycetaceae* 1.76%, *Porphyromonadaceae* 1.50%, *Carnobacteriaceae* 1.06%, and others at 2.51%. A comparison of the top 20 oral microbial families in PTB vs. TB groups is shown in Figure 3A. At the genus level, after removing the unknown genera, the dominant members were *Streptococcus*, representing 40.83%, followed by *Veillonella* 11.39%, *Prevotella* 7.0%, *Hemophilus* 5.89%, *Neisseria* 5.65%, *Rothia* 4.65%*, Gemella* 4.20%, *Bergeyella* 2.45%, *Fusobacterium* 2.39%, and *Leptotrichia* 1.87%. A comparison of the top 20 oral microbial genera and species between PTB and TB groups are shown in Figures 3B,C, respectively. At the species level *S. anginosus, S. cristatus, S. infantis, S. mitis, S. oralis, S. pneumoniae, S. sanguinis, S. timonensis* were the most abundant.

### 3.4. Differential relative abundance between birth groups

**Figure 4:**
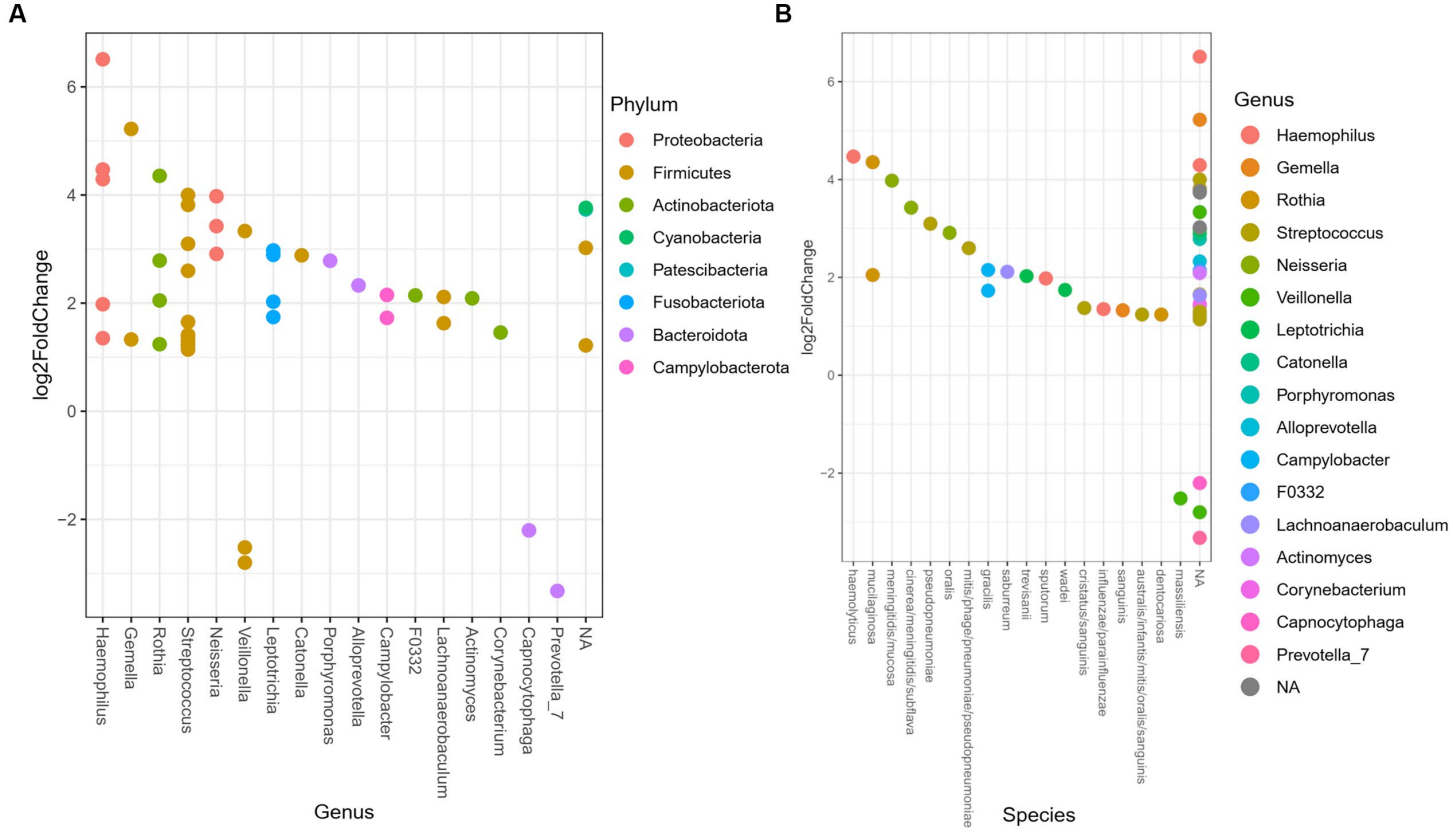
Differential relative abundance of oral bacteria in term vs. preterm delivery groups. **(A)** Differential relative abundance of phyla, genera, and **(B)** species in log2fold change between the oral microbiome of the term birth (TB) and preterm birth (PTB) delivery groups.

The differential relative abundance analysis identified 48 ASV (Supplementary Table 1), that could be assigned 17 genera. The log2fold change between the genera in the oral microbiome of the TB group vs. the PTB group is shown in Figure 4A. The TB group had a more than twofold differential relative decrease of genera *Prevotella, Capnocytophaga*, and *Veillonella*, compared to PTB group. We further analyzed the differential relative abundance of genera and species and observed that the relative abundance of *Haemophilus haemolyticus* and *Neisseria* spp.; *N. oralis, N. cinerea, N. meningitidis, N. subflava*, and *N. mucosa* were up to fourfold higher in the TB group compared with the PTB group (Figures 4A,B). The differential relative abundance of the genus *Leptotricha*, was also more than twofold higher in the oral microbiome of the TB group compared to the PTB group (Figure 4A). Similarly, *Porphyromonas* spp. (Figure 4B), was more present in the TB group compared to the PTB group. But interestingly, specifically *Porphyromonas gingivalis*, a species previously linked to PTB (10) was not statistically significant when comparing gestational groups. At the species level, the highest differential relative abundance of *Veillonella massiliensis* were present in the PTB group compared with the TB group.

## 4. Discussion

We investigated the characteristics of the oral microbiome in PTB compared to TB by sequencing the V3-V4 region of the 16S rRNA gene on the largest number of pregnant women reported so far. We included 152 participants, of which 61 delivered preterm at up to 36 6/7 weeks of gestation and 91 delivered at term at or after 38 0/7 weeks of pregnancy.

Our analysis showed high oral microbiome diversity in pregnant women in line with previously reported general oral microbiome diversity in other groups (27). The top identified taxa were concordant with previously reported oral microbiome compositions during pregnancy (9, 28). A recent report by Ye et al. (29) analyzing how oral microbiome correlates with low birth weight pregnancy, showed a similar levels of richness and diversity of oral microbiome were observed in both groups of pregnant women.

In our study, *Firmicutes* and *Bacteroidetes* were more abundant in women with a PTB than in women with a TB, while *Proteobacteria* were less prevalent in women with a PTB (Figure 4). In case of *Proteobacteria* this is expected as they are generally associated with oral health (30). These results are also concordant with a previous study using 16S rRNA gene amplicon sequencing, that compared the blood microbial communities in PTB compared to TB (7). Similar findings were also observed in a study comparing the oral microbiome in association with PTB between pregnant women with gingivitis compared to the healthy oral pregnancy by sequencing the 16S rRNA (31).

Some previous reports have suggested that microbes from the oral cavity can be transmitted to the pregnant uterus via blood (32, 33). In both reports, using the 16S rRNA method, the same strain was detected in the uterus and maternal oral cavity but not in the vaginal sample. This theory is supported by other studies detecting blood microbiome in pregnant women, where most of the enriched bacteria were annotated as oral bacteria (7), (34, p. 20). According to 16S rRNA gene amplicon sequencing, blood microbial communities during pregnancy showed results at the phylum level concordant with our oral microbiome findings: *Firmicutes* and *Bacteroidetes* were more abundant in women with a PTB than in women with a TB, while *Proteobacteria* was less prevalent in women with a PTB (29). However, at the genus level, there was a less clear overlap with our results likely reflecting interpersonal subject differences (7).

Historically, many previous studies have linked oral disease to PTB (10, 30, 31, 35–37). While one of the earliest studies reported diagnosed periodontal disease in as much as 83% of PTB and only in 20% of TB cases (35), newer studies conducted using 16S rRNA gene sequencing show similar results to our study in regard to phylum level composition, as well as alpha and beta diversity, despite several differences between our cohorts (31). Indeed, recent studies examining the oral microbiome in the context of periodontal disease found alpha-diversity to be significantly affected by PTB, likely reflecting the overrepresentation of certain bacteria, or dysbiotic bacterial shift, due to the infection (30, 38). Our results are therefore expected as we have excluded subjects taking antibiotic therapy for any type of infection. A recently published meta-analysis of 12 studies examining the association between the oral microbiome and PTB (9) suggested that high levels of the same periodontal pathogens during pregnancy were evidently associated with an increased risk for PTB. However, most of these previous studies analyzed pre-selected oral pathogenic bacteria, such as *Porphyromonas gingivalis, Aggregatibacter actinomycetemcomitans, Tannerella forsythia, Treponema denticola, Fusobacterium nucleatum*, and *Prevotella intermedia* (9). Similarly, several studies, looking at periodontosis and PTB, have reported that major periodontal pathogen bacteria such as *Fusobacterium nucleatum, Treponema denticola*, and *Porphyromonas gingivalis* (10–12) are present in the placenta and connected with PTB, however the differences between these individual studies in terms of approach and cohorts makes them hard to compare directly. For similar reasons, it is difficult to assess direct contribution of specific oral pathogen bacteria as causative agents of PTB.

Unlike previous studies, our study focused exclusively on the oral microbiome in PTB, comparing our otherwise healthy cohort with a well-matched control group (we utilized strict inclusion criteria, meaning that PTB due to most maternal or fetal complications, such as preeclampsia or fetal anomalies, etc., were excluded from the study, as well as those women taking antibiotic therapy for any reason). Furthermore, another strength of our study is the homogeneity of the study population. All participants were Caucasian and belonged to the same Slavic ethnicity, reducing the bias that can result from cultural dietary differences, etc. In regard to the limitations, our sampling was conducted at a single time, which was upon admission to the hospital. This resulted in different gestation ages at sampling; however, previous studies have showed that the oral microbiome remains stable during the course of pregnancy (32, 39), making this less likely to affect our results. Furthermore, the collection time logistical limitations prevented formal assessment of the oral health of the subjects. Because of this as well as very high prevalence of gingivitis overall in healthy pregnancy, our approach was to exclude all cases requiring medical attention by excluding all women who had used antibiotics for any type of infection during the week before the inclusion to the hospital. In this way, and as is apparent also by our final data (not showing periodontitis-associated pathogens), we believe we have successfully excluded any cases of periodontitis infection from our cohort. Finally, a possible limitation of our study is the use of marker gene sequencing that may have affected species and strain level identification.

When looking at the previously reported associations between genera/species present in the oral microbiome and PTB (10, 31, 37, 40) we observed the differential relative abundance of *Fusobacterium* phylum, genus *Leptotricha*, was more than twofold higher in the oral microbiome of the TB group compared to the PTB group, and that the genera *Veillonella, Prevotella*, and *Capnocytophaga* were increased in the PTB group (Figure 4A). Similarly, *Porphyromonas* spp. (Figure 4B), was more present in the TB group compared to the PTB group. But interestingly, specifically *Porphyromonas gingivalis* was not statistically significant when comparing gestational groups in our study, likely reflecting the difference in the cohorts. And indeed, similarly to what has been shown in the most recent review of studies with similar design (9), our results do not show a significant association of previously shown pathogens such as *Porphyromonas gingivalis* when comparing gestational groups.

Of the genera identified as enriched in the PTB group by our study, *Capnocytophaga* has previously been implicated in preterm birth, and as a risk factor for chorioamnionitis and neonatal infection (41), while our observation of the enrichment of *Veillonella massiliensis* in the PTB group compared with the TB group has, to the best of our knowledge, been observed for the first time. While the genus *Veillonella* is part of the normal oral, gastrointestinal, respiratory and urogenital microbiome, the species *Veillonella massiliensis* was first isolated from two human colostrum samples from two different mothers in 2016 (42) and has not yet been linked with human disease.

## 5. Conclusion

In conclusion, we identified the dominant microorganisms at the phylum level in the oral microbiome of all pregnant women regardless of birth week outcomes as belonging to phyla *Firmicutes, Proteobacteria, Bacteroidetes, Fusobacteria*, and *Actinobacteria*.

The phyla *Firmicutes* and *Bacteroidetes* were relatively more abundant in women with a PTB than in women with a TB, while *Proteobacteria* was less prevalent in women with a PTB. At the genus level, *Veillonella*, *Prevotella*, and *Capnocytophaga* were enriched in the PTB, and on the species level, only *Veillonella massillensis* was shown to be increased in the PTB group.

Our results suggest taxa such as *Veillonella*, *Prevotella*, and *Capnocytophaga* in the maternal oral microbiome are associated with PTB independently of clinically apparent infection, uterine anomalies, and other pregnancy complications. The clarification of the role of those taxa in the etiology of PTB merits further research.

## Data availability statement

The datasets presented in this study can be found in online repositories. The names of the repository/repositories and accession number(s) can be found at: https://www.ncbi.nlm.nih.gov/, PRJNA913792.

## Ethics statement

The studies involving human participants were reviewed and approved by the Slovenian National Medical Ethics Committee, Ministry of Health, Ljubljana, Slovenia (approval number 90/02/15). The participants provided their written informed consent to participate in this study. The studies were conducted in accordance with the local legislation and institutional requirements.

## Author contributions

TP-S, BP, and MVŠ conceived and designed the study. MVŠ, TP-S, and KH collected the samples and data and analyzed data from the patients. AK, ANZ, and AM performed sequencing experiments and analyzed the data. MVŠ and AK wrote the manuscript. All authors contributed to the article and approved the submitted version.

## Funding

This study was funded by the Slovenian Research Agency (ARRS), grant no. P3-0326 and the University Medical Centre Ljubljana, grant no. 20170068.

## Conflict of interest

The authors declare that the research was conducted without any commercial or financial relationships that could be construed as a potential conflict of interest.

## Publisher’s note

All claims expressed in this article are solely those of the authors and do not necessarily represent those of their affiliated organizations, or those of the publisher, the editors and the reviewers. Any product that may be evaluated in this article, or claim that may be made by its manufacturer, is not guaranteed or endorsed by the publisher.

## References

[1] Blencowe H Cousens S Oestergaard MZ Chou D Moller A-B Narwal R. National, regional, and worldwide estimates of preterm birth rates in the year 2010 with time trends since 1990 for selected countries: a systematic analysis and implications. Lancet. (2012) 379:2162–72. doi: 10.1016/S0140-6736(12)60820-4, PMID: 22682464

[2] Vogel JP Chawanpaiboon S Moller A-B Watananirun K Bonet M Lumbiganon P. The global epidemiology of preterm birth. Best Pract Res Clin Obstet Gynaecol. (2018) 52:3–12. doi: 10.1016/j.bpobgyn.2018.04.003 29779863

[3] Mysorekar IU Cao B. Microbiome in parturition and preterm birth. Semin Reprod Med. (2014) 32:050–5. doi: 10.1055/s-0033-1361830 24390921

[4] Vinturache AE Gyamfi-Bannerman C Hwang J Mysorekar IU Jacobsson B. Maternal microbiome—a pathway to preterm birth. Semin Fetal Neonatal Med. (2016) 21:94–9. doi: 10.1016/j.siny.2016.02.004 26936188

[5] Fitzpatrick A Venugopal K Scheil W McDonald SP Jesudason S. The Spectrum of adverse pregnancy outcomes based on kidney disease diagnoses: a 20-year population study. Am J Nephrol. (2019) 49:400–9. doi: 10.1159/000499965, PMID: 30982041

[6] Mohanty T Doke PP Khuroo SR. Effect of bacterial vaginosis on preterm birth: a meta-analysis. Arch Gynecol Obstet. (2022). doi: 10.1007/s00404-022-06817-5, PMID: 36251068

[7] You Y-A Yoo JY Kwon EJ Kim YJ. Blood microbial communities during pregnancy are associated with preterm birth. Front Microbiol. (2019) 10:1122. doi: 10.3389/fmicb.2019.01122, PMID: 31214131 PMC6558066

[8] Manrique-Corredor EJ Orozco-Beltran D Lopez-Pineda A Quesada JA Gil-Guillen VF Carratala-Munuera C. Maternal periodontitis and preterm birth: systematic review and meta-analysis. Community Dent Oral Epidemiol. (2019) 47:243–51. doi: 10.1111/cdoe.12450, PMID: 30812054

[9] Jang H Patoine A Wu TT Castillo DA Xiao J. Oral microflora and pregnancy: a systematic review and meta-analysis. Sci Rep. (2021) 11:16870. doi: 10.1038/s41598-021-96495-1, PMID: 34413437 PMC8377136

[10] Hasegawa-Nakamura K Tateishi F Nakamura T Nakajima Y Kawamata K Douchi T. The possible mechanism of preterm birth associated with periodontopathic *Porphyromonas gingivalis*. J Periodontal Res. (2011) 46:497–504. doi: 10.1111/j.1600-0765.2011.01366.x, PMID: 21488875

[11] Ye C Katagiri S Miyasaka N Kobayashi H Khemwong T Nagasawa T. The periodontopathic bacteria in placenta, saliva and subgingival plaque of threatened preterm labor and preterm low birth weight cases: a longitudinal study in Japanese pregnant women. Clin Oral Investig. (2020a) 24:4261–70. doi: 10.1007/s00784-020-03287-4, PMID: 32333174

[12] Ye C Kobayashi H Katagiri S Miyasaka N Takeuchi Y Kuraji R. The relationship between the anti-*Porphyromonas gingivalis* immunoglobulin G subclass antibody and small for gestational age delivery: a longitudinal study in pregnant Japanese women. Int Dent J. (2020b) 70:296–302. doi: 10.1111/idj.12548, PMID: 32185796 PMC9379198

[13] Committee on Obstetric Practice American Institute of Ultrasound in Medicine Society for Maternal–Fetal Medicine. Committee Opinion No 700: Methods for Estimating the Due Date. Obstet Gynecol. (2017) 129:e150–4. doi: 10.1097/AOG.0000000000002046 28426621

[14] Hočevar K Maver A Vidmar Šimic M Hodžić A Haslberger A Premru Seršen T. Vaginal microbiome signature is associated with spontaneous preterm delivery. Front Med. (2019) 6:201. doi: 10.3389/fmed.2019.00201, PMID: 31552254 PMC6746969

[15] Ravel J Gajer P Abdo Z Schneider GM Koenig SSK McCulle SL. Vaginal microbiome of reproductive-age women. Proc Natl Acad Sci U S A. (2011) 108:4680–7. doi: 10.1073/pnas.1002611107, PMID: 20534435 PMC3063603

[16] Callahan BJ Sankaran K Fukuyama JA McMurdie PJ Holmes SP. Bioconductor workflow for microbiome data analysis: from raw reads to community analyses. F1000 Res. (2016b) 5:1492. doi: 10.12688/f1000research.8986.1 PMC4955027 27508062

[17] Callahan BJ McMurdie PJ Rosen MJ Han AW Johnson AJA Holmes SP. DADA2: high-resolution sample inference from Illumina amplicon data. Nat Methods. (2016a) 13:581–3. doi: 10.1038/nmeth.3869, PMID: 27214047 PMC4927377

[18] Callahan BJ McMurdie PJ Holmes SP. Exact sequence variants should replace operational taxonomic units in marker-gene data analysis. ISME J. (2017) 11:2639–43. doi: 10.1038/ismej.2017.119, PMID: 28731476 PMC5702726

[19] Callahan Benjamin (2020). RDP taxonomic training data formatted for DADA2 (RDP trainset 18/release 11.5). *Zenodo* doi: 10.5281/ZENODO.4310151

[20] McLaren MR Callahan BJ. (2021). Silva 138.1 prokaryotic SSU taxonomic training data formatted for DADA2. Zenodo doi: 10.5281/ZENODO.4587955

[21] McMurdie PJ Holmes S. Phyloseq: an R package for reproducible interactive analysis and graphics of microbiome census data. PLoS One. (2013) 8:e61217. doi: 10.1371/journal.pone.0061217, PMID: 23630581 PMC3632530

[22] Oksanen J. Simpson G. L. Blanchet F. G. Kindt R. Legendre P. Minchin P. R.. Vegan: community ecology package. (2022). Available at: https://CRAN.R-project.org/package=vegan

[23] Love MI Huber W Anders S. Moderated estimation of fold change and dispersion for RNA-seq data with DESeq2. Genome Biol. (2014) 15:550. doi: 10.1186/s13059-014-0550-8 25516281 PMC4302049

[24] Cameron ES Schmidt PJ Tremblay BJM Emelko MB Müller KM. Enhancing diversity analysis by repeatedly rarefying next generation sequencing data describing microbial communities. Sci. Rep. (2021) 11:22302. doi: 10.1038/s41598-021-01636-1 34785722 PMC8595385

[25] Wickham H. Ggplot2: Elegant graphics for data analysis. New York: Springer-Verlag (2016).

[26] Glover AV Manuck TA. Screening for spontaneous preterm birth and resultant therapies to reduce neonatal morbidity and mortality: a review. Semin Fetal Neonatal Med. (2018) 23:126–32. doi: 10.1016/j.siny.2017.11.007, PMID: 29229486 PMC6381594

[27] Deo PN Deshmukh R. Oral microbiome: unveiling the fundamentals. J Oral Maxillofac Pathol JOMFP. (2019) 23:122–8. doi: 10.4103/jomfp.JOMFP_304_18, PMID: 31110428 PMC6503789

[28] Saadaoui M Singh P Al Khodor S. Oral microbiome and pregnancy: a bidirectional relationship. J Reprod Immunol. (2021) 145:103293. doi: 10.1016/j.jri.2021.103293, PMID: 33676065

[29] Ye C You M Huang P Xia Z Radaic A Tang J. Clinical study showing a lower abundance of Neisseria in the oral microbiome aligns with low birth weight pregnancy outcomes. Clin Oral Investig. (2022) 26:2465–78. doi: 10.1007/s00784-021-04214-x, PMID: 34622310 PMC8898250

[30] Bartha V Steinmacher S Wittlinger R Boutin S Pauluschke-Fröhlich J von Ohle C. Gain a baby lose a tooth—is there an association between periodontitis and preterm birth? J Clin Med. (2022) 11:7183. doi: 10.3390/jcm11237183, PMID: 36498757 PMC9739114

[31] Yang I Knight AK Dunlop AL Corwin EJ. Characterizing the subgingival microbiome of pregnant African American women. J Obstet Gynecol Neonatal Nurs. (2019) 48:140–52. doi: 10.1016/j.jogn.2018.12.003, PMID: 30597136 PMC7252202

[32] Gonzales-Marin C Spratt DA Allaker RP. Maternal oral origin of *fusobacterium nucleatum* in adverse pregnancy outcomes as determined using the 16S-23S rRNA gene intergenic transcribed spacer region. J Med Microbiol. (2013) 62:133–44. doi: 10.1099/jmm.0.049452-0, PMID: 23002071

[33] Han YW Redline RW Li M Yin L Hill GB McCormick TS. *Fusobacterium nucleatum* induces premature and term stillbirths in pregnant mice: implication of Oral Bacteria in preterm birth. Infect Immun. (2004) 72:2272–9. doi: 10.1128/IAI.72.4.2272-2279.2004, PMID: 15039352 PMC375172

[34] Yin C Chen J Wu X Liu Y He Q Cao Y. Preterm birth is correlated with increased Oral originated microbiome in the gut. Front Cell Infect Microbiol. (2021) 11:579766. doi: 10.3389/fcimb.2021.579766, PMID: 34222033 PMC8248533

[35] Dörtbudak O Eberhardt R Ulm M Persson GR. Periodontitis, a marker of risk in pregnancy for preterm birth. J Clin Periodontol. (2005) 32:45–52. doi: 10.1111/j.1600-051X.2004.00630.x, PMID: 15642058

[36] Lin D Moss K Beck JD Hefti A Offenbacher S. Persistently high levels of periodontal pathogens associated with preterm pregnancy outcome. J Periodontol. (2007) 78:833–41. doi: 10.1902/jop.2007.060201, PMID: 17470016

[37] Usin MM Tabares SM Parodi RJ Sembaj A. Periodontal conditions during the pregnancy associated with periodontal pathogens. J Investig Clin Dent. (2013) 4:54–9. doi: 10.1111/j.2041-1626.2012.00137.x, PMID: 23335585

[38] Yang I Claussen H Arthur RA Hertzberg VS Geurs N Corwin EJ. Subgingival microbiome in pregnancy and a potential relationship to early term birth. Front Cell Infect Microbiol. (2022) 12:873683. doi: 10.3389/fcimb.2022.873683, PMID: 35646730 PMC9132049

[39] DiGiulio DB Callahan BJ McMurdie PJ Costello EK Lyell DJ Robaczewska A. Temporal and spatial variation of the human microbiota during pregnancy. Proc Natl Acad Sci U S A. (2015) 112:11060–5. doi: 10.1073/pnas.1502875112, PMID: 26283357 PMC4568272

[40] Cassini MA Pilloni A Condò SG Vitali LA Pasquantonio G Cerroni L. Periodontal bacteria in the genital tract: are they related to adverse pregnancy outcome? Int J Immunopathol Pharmacol. (2013) 26:931–9. doi: 10.1177/039463201302600411, PMID: 24355228

[41] Lopez E Raymond J Patkai J El Ayoubi M Schmitz T Moriette G. Capnocytophaga species and preterm birth: case series and review of the literature. Clin Microbiol Infect. (2010) 16:1539–43. doi: 10.1111/j.1469-0691.2009.03151.x, PMID: 20041890

[42] Togo AH Des Robert C Bonnet M Fournier P-E Raoult D Million M. «Veillonella massiliensis», a new anaerobic species isolated from human colostrum. Hum Microbiome J. (2017) 4:20–1. doi: 10.1016/j.humic.2017.05.003

